# Graphene Oxide affects *Staphylococcus aureus* and *Pseudomonas aeruginosa* dual species biofilm in Lubbock Chronic Wound Biofilm model

**DOI:** 10.1038/s41598-020-75086-6

**Published:** 2020-10-28

**Authors:** Mara Di Giulio, Silvia Di Lodovico, Antonella Fontana, Tonino Traini, Emanuela Di Campli, Serena Pilato, Simonetta D’Ercole, Luigina Cellini

**Affiliations:** 1 Department of Pharmacy, University “G. d’Annunzio” Chieti-Pescara, Via dei Vestini, 31, 66100 Chieti, Italy; 2 Department of Medical Oral and Biotechnological Sciences, University “G. d’Annunzio” Chieti-Pescara, Via dei Vestini, 31, 66100 Chieti, Italy

**Keywords:** Microbiology, Bacteria, Biofilms, Experimental models of disease

## Abstract

Chronic wound management becomes a complex procedure because of the persistence of forming biofilm pathogens that do not respond to antimicrobial treatment. The aim of this paper is to detect the Graphene Oxide-GO effect on *Staphylococcus aureus* and *Pseudomonas aeruginosa* dual species wound biofilm in Lubbock Chronic Wound Biofilm-LCWB model. LCWB is a recognized model that mimics the spatial microbial colonization into chronic wounds and reproduces the wound and its clot. *Staphylococcus aureus* PECHA 10 and *P. aeruginosa* PECHA 4, are the pathogens used in the study. The GO effect on both in forming and mature biofilms, is detected by the evaluation of the CFU/mg reduction, the cell viability and ultrastructural analysis of the treated LCWBs. Graphene Oxide, at 50 mg/l, shows a significant antibiofilm effect in forming and mature LCWBs. In particular, during the biofilm formation, GO reduces the *S. aureus* and *P. aeruginosa* growth of 55.05% ± 4.73 and 44.18% ± 3.91 compared to the control. In mature biofilm, GO affects *S. aureus* and *P. aeruginosa* by reducing their growth of 70.24% ± 4.47 and 63.68% ± 17.56, respectively. Images taken by SEM show that GO display a disaggregated microbial effect also disrupting the fibrin network of the wound-like biofilm framework. In conclusion, GO used against microorganisms grown in LCWB, displays a significant inhibitory action resulting in a promising tool for potential application in wound management.

## Introduction

Wounds that do not respond normally to treatments after four weeks or do not heal completely within two months are defined as chronic wounds. Today, chronic wounds represent a silent epidemic problem affecting billions of people worldwide^1^ causing pain, prolonged hospital stays, depression and discomfort, reducing, in general, the quality of life in patients^2,3^.

The wound healing process is extremely complex, representing a regulated interplay among many host factors^4^, including microbial infections. In particular, bacterial colonization/infection is considered as a primary cause of chronic inflammation. Moreover, the ‘critical level’ of microbial concentration that defines colonization or clinically relevant infection is affected by the microbial capability to proliferate in polymicrobial biofilm that may significantly delay the wound healing process^5^.

*Staphylococcus aureus* and *Pseudomonas aeruginosa* are the prevailing bacterial species that co-infect chronic wounds, in up to 60% of cases^6,7^_._ In the first step of colonization, their relationship is competitive, then, becomes synergistic^5^ and the co-infection provides mutual benefit between species. In general, a first goal of polymicrobial biofilms in chronic wound infections is related to the increased antimicrobial tolerance compared with mono species biofilms, resulting in a greater persistence of colonization^8^.

In particular, dual species biofilm including *S. aureus* and *P. aeruginosa* makes them more refractory to the treatment due to an increase of the structural integrity of the produced biofilm^5^ resulting in a delay of wound healing that represents a key virulence factor in the wound chronicization^9^.

These considerations underline the difficulty of chronic wound management, strongly suggesting the need of research of novel treatment strategies aimed to disrupt the produced microbial biofilms.

In the last 15 years, graphene and graphene derivatives have emerged as materials with exceptional physical and chemical characteristics of interest for meaningful applications. In particular, for biomedical applications, hydrophilic graphene derivatives, such as Graphene Oxide (GO), have been prevailingly used and tested. Similarly, to graphene, Graphene Oxide is a one atom thick molecule and presents a high aspect ratio, i.e. a high surface compared to the relevant weight.

Differently from graphene, consisting essentially of sp^2^ carbon atoms and strong covalent Csp^2^–Csp^2^ bonds, GO presents oxygenated carbon moieties that confer it a low tendency to form aggregates and a strong capability to disperse homogeneously in water^10^ and other polar environments. Thanks to van der Waals, electrostatic, π–π and hydrogen bond interactions, GO can easily adsorb polymers and proteins^11^, negatively interfering with the growth of the microorganisms and their aggregation and adhesion to surfaces^12^. These interesting recognized GO properties represent a promising approach for antimicrobial and antibiofilm applications.

Recently, GO has been reported to exhibit good antibacterial activity toward both Gram positive and Gram negative bacteria^13^ together with antibiofilm properties against human pathogens^14–18^ and, in particular, chronic wound microbial isolates^12^.

The Lubbock Chronic Wound Biofilm (LCWB) model is the first in vitro model mimicking a realistic in vivo multispecies biofilm that develops into chronic wounds. This model, easily allows the biofilm growth of a multispecies bacterial population such as *S. aureus* and *P. aeruginosa*^18^.

The *S. aureus* coagulase activity produces an insoluble fibrin network that designs and arranges the wound-like biofilm framework representing a scaffold on which bacteria can adhere. This adhesion favours the development of a tridimensional biofilm that interconnects bacteria to each other and reproduces faithfully the spatial microbial colonization of the chronic wound.

Then, the LCWB model is transferred into an artificial wound bed allowing the study of the effect of novel wound dressings^19^.

The aim of this work is to evaluate the effect of GO on *S. aureus* and *P. aeruginosa* dual species biofilm in LCWB model both in forming and on mature biofilm.

## Results

Graphene Oxide dispersions at the concentration used in the experiments are characterized by using Dynamic Laser Light Scattering in terms of dimensions and polidispersity (Table S1). Diameters of 598.3 ± 10.3 nm and 668.1 ± 33.7 nm are obtained at 25 and 37 °C, respectively, thus confirming the micrometric characterization reported by Graphenea. Moreover, the polidispersity of 0.261 ± 0.019 and 0.254 ± 0.013 is indicative, as expected, of non-perfectly homogenous samples.

**Figure 1:**
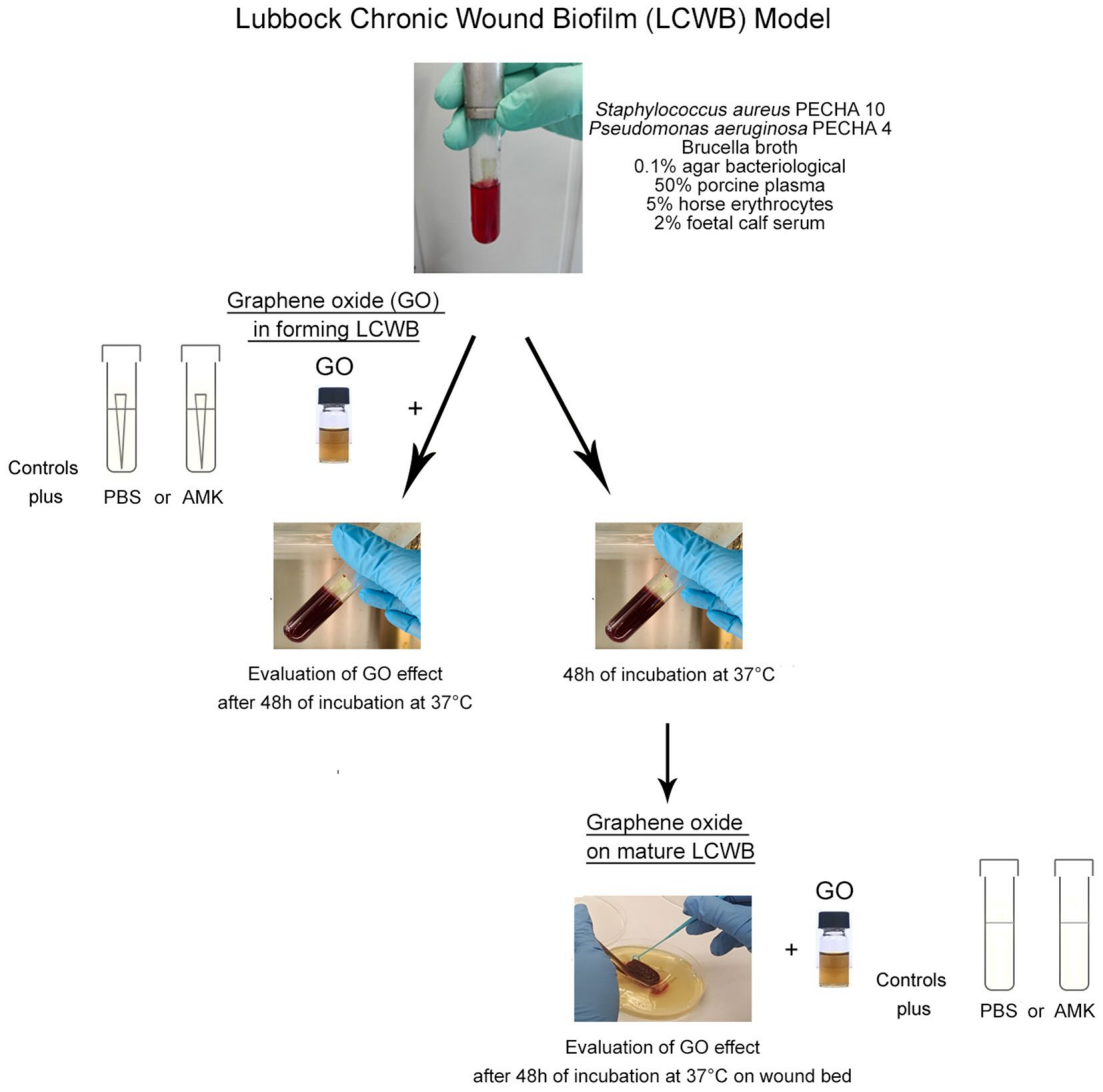
Experimental plan performed in the study. The LCWB was prepared combining 10 µl of *S. aureus* PECHA 10 and 10 µl of *P. aeruginosa* PECHA 4 at the final concentration of 10^6^ CFU/ml and 10^5^ CFU/ml, respectively into glass tubes with a sterile pipette tip containing a special medium composed of Brucella Broth, 0.1% agar bacteriological, 50% porcine plasma, 5% horse erythrocytes and 2% Foetal Calf serum (top). The GO effect, in forming LCWB, was evaluated by adding to the aforementioned mixture, 100 µl of GO at a concentration of 2.5 mg/ml; 100 µl of PBS or 100 µl of AMK (at a final concentration of 8 mg/l) were included in the control samples. The test tubes were analysed after incubation of 48 h at 37 °C (center left). The effect of GO on mature LCWB was evaluated at 48 h of incubation, harvesting it from glass tube after removing the pipette tip. The GO, at the same concentration, was added to the mature LCWB that was placed on Petri dish containing Bolton Broth with 1.5% agar bacteriological to produce the “wound bed”; PBS or AMK (at a final concentration of 64 mg/l) were included in the control samples. The treated mature LCWBs were analysed after incubation of 48 h at 37 °C (bottom right).

Figure 1 displays graphically the followed experimental plan for the evaluation of the effect of GO on *S. aureus* and *P. aeruginosa* dual species biofilm in forming and mature LCWB.

Graphene Oxide is capable to inhibit the growth of clinical isolates from chronic wounds *S. aureus* PECHA 10 and *P. aeruginosa* PECHA 4 dual species biofilm in LCWB model.

**Figure 2:**
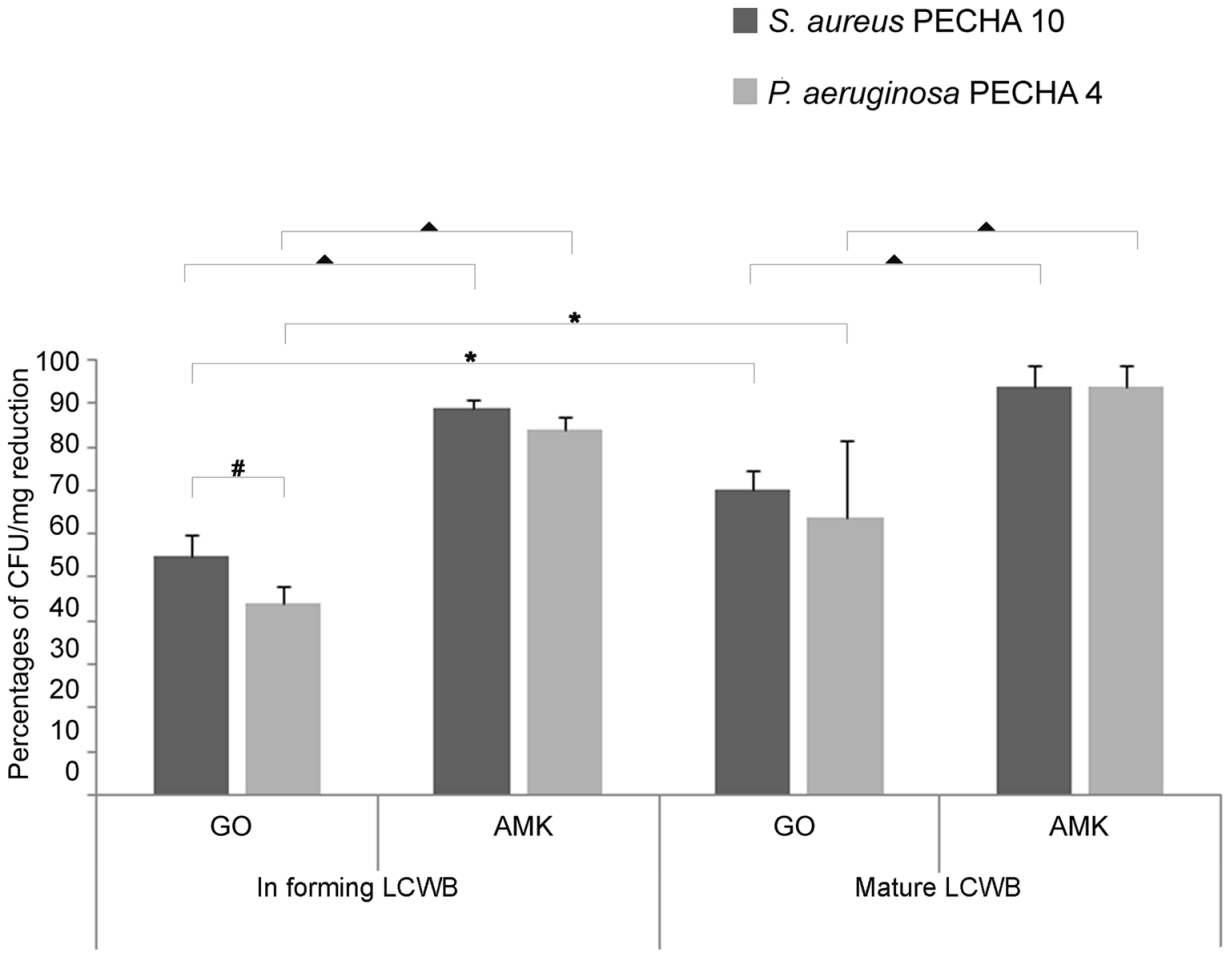
Percentages of CFU/mg reduction of *S. aureus* PECHA 10 and *P. aeruginosa* PECHA 4 in dual species in forming and mature LCWBs in presence of GO and AMK. All data are statistically significant in respect to the controls (*p* < 0.05). ^▲^Statistically significant (*p* < 0.05) value between GO and AMK in forming and mature LCWBs for each strain. *Statistically significant (*p* < 0.05) value between in forming and mature LCWBs, for each strain in each tested condition. ^#^Statistically significant (*p* < 0.05) value between *S. aureus* PECHA 10 and *P. aeruginosa* PECHA 4 in each tested condition.

Figure 2 shows the percentage of CFU/mg biofilm reduction after treatment with GO and Amikacin (AMK) both in forming and on mature biofilms compared with the untreated samples taking into account each LCWB weight. In Table S2 reported are the mean LCWB weight and bacterial load CFUs/LCWB mg values of untreated and GO/AMK treated in forming and mature LCWBs. In each tested condition, GO displays a significant antibiofilm effect against *S. aureus* PECHA 10 (*p* < 0.001) and *P. aeruginosa* PECHA 4 (*p* < 0.001) in respect to in forming and mature biofilm controls, respectively. The addition of 50 mg/l GO, during the dual species *S. aureus* PECHA 10 and *P. aeruginosa* PECHA 4 in forming LCWB, reduces the microbial growth (55.05% ± 4.73 and 44.18% ± 3.91 of growth reduction, respectively) in respect to the controls. These data are compared with the percentages of biofilm reduction of 89.08% ± 1.86 and 84.14% ± 2.66 in presence of 8 mg/l of AMK that is the sub-MIC value of each detected microorganism (*S. aureus* PECHA 10, MIC AMK = 16 mg/l; *P. aeruginosa* PECHA 4, MIC AMK = 32 mg/l) (*p* < 0.001).

Regarding the mature LCWB, GO expresses a very interesting biofilm growth reduction of 70.24% ± 4.47 (*p* < 0.001) and 63.68% ± 17.56 (*p* < 0.001) for *S. aureus* PECHA 10 and *P. aeruginosa* PECHA 4, respectively. In presence of a concentration of AMK (64 mg/l) greater than the MIC values indicated previously for each detected microorganism, the percentages of CFU/mg reduction are 93.60% ± 5.18 and 93.73% ± 8.28 for *S. aureus* PECHA 10 and *P. aeruginosa* PECHA 4, respectively. In each experimental assay, the best effect of GO in dual species biofilm is detected against *S. aureus* PECHA 10 compared with *P. aeruginosa* PECHA 4, although a statistical significance is obtained only in forming LCWB (*p* = 0.0042).

**Figure 3:**
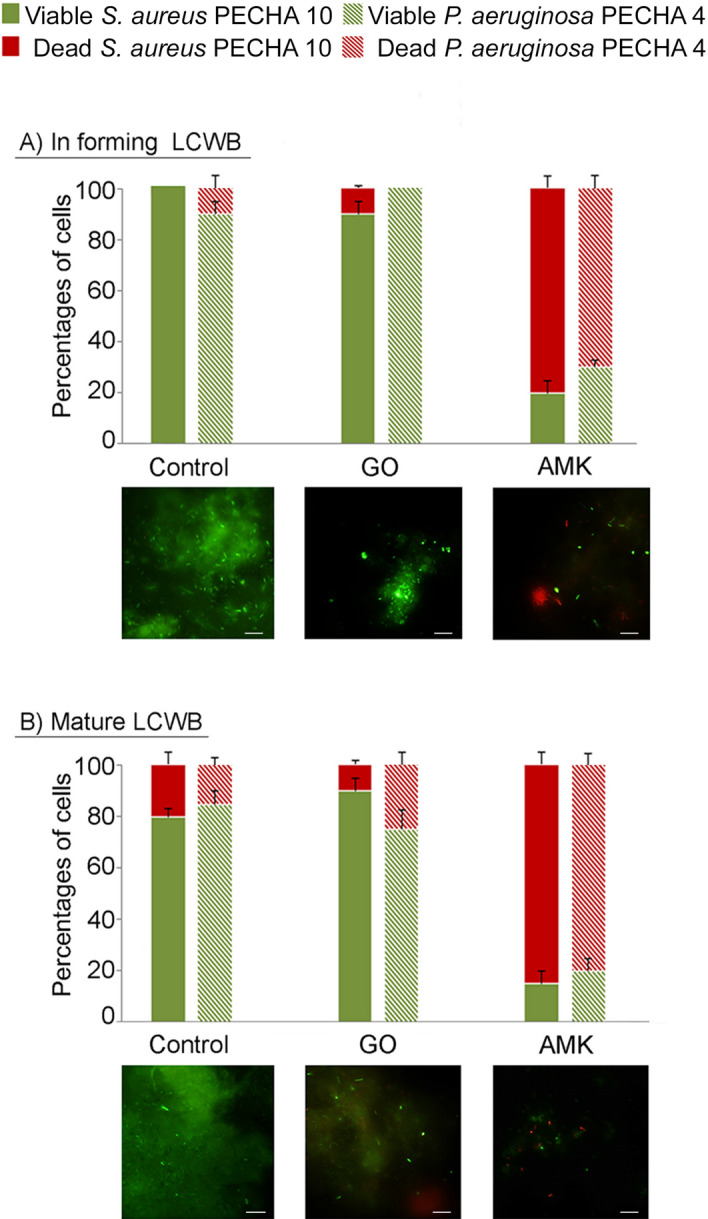
Percentages of *S. aureus* PECHA 10 and *P. aeruginosa* PECHA 4 viability in dual species in forming (**A**) and mature (**B**) LCWBs in presence of GO and AMK. For each detection, representative Live/Dead images of in forming and mature LCWBs in presence of GO and AMK compared with the controls, are shown. Original magnification, 1000 × . In Live/Dead images, sessile population in biofilms with a damaged membrane (dead cells) is stained in red, whereas viable cells are green stained. The images observed at fluorescent Leica 4000 DM microscopy are recorded at an excitation/emission wavelengths of 485/498 nm for SYTO 9 and of 535/617 nm for Propidium iodide.

The evaluation of GO effect on the viability of the examined microbial population in the produced biofilms demonstrates its prevailing bacteriostatic effect (Fig. 3). In fact, 90% of viable coccoid cells are detected both in forming and mature biofilms and 100% and 75% of viable rod bacteria are identified in forming and mature biofilms, respectively (Fig. 3A,B).

Representative Live/Dead images for each detected condition are also shown in Fig. 3. In all treated samples, images clearly display scattered cells with prevalent green bacteria in GO treated samples and a major number of red bacteria in samples treated with AMK.

**Figure 4:**
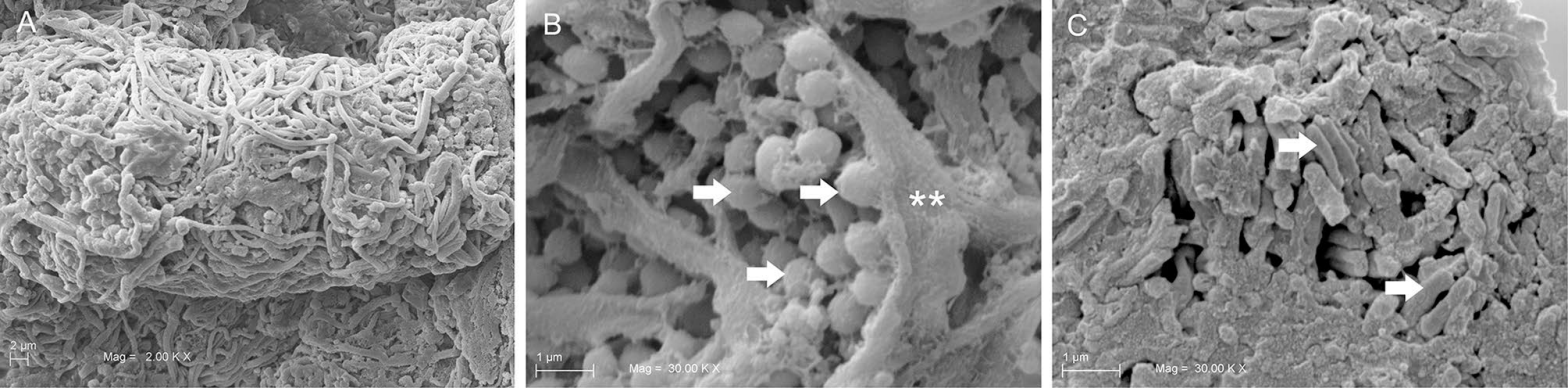
SEM micrographs of mature LCWB untreated specimen. In (**A**), at low magnification (2 K ×) a dense fibrin network is present on the specimen surface; at higher magnification (30 K ×) the microbial species appear to be spatially separated. In (**B**), *S. aureus* PECHA 10 cells (arrows) appear to be wrapped by a fibrin cup (asterisks). In (**C**), bacillary *P. aeruginosa* PECHA 4 cells (arrows) are grouped to each other in a superficial area of the specimen.

The Scanning Electron Microscopy (SEM) images display an unmixed spatial distribution of bacterial species in the untreated mature LCWB (Fig. 4). In particular, Fig. 4A shows the thick and clustered structure of the untreated sample with a complex interconnected fibrous network. With major magnification, Fig. 4B,C clearly highlights the single microbial species location with coccoid *S. aureus* PECHA 10 cells (Fig. 4B) grouped separately to bacillary *P. aeruginosa* PECHA 4 cells (Fig. 4C). Interestingly, this separated spatial microbial distribution is detected in all observed sections (not shown).

**Figure 5:**
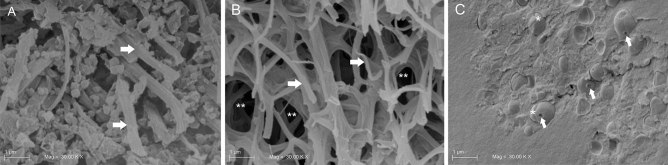
SEM micrographs of mature LCWBs on the fracture wall at 30 K × of magnifications. In (**A**), an untreated specimen with a denser fibrin network (white arrows); in (**B**), a GO treated specimen with loose fibrin network (arrows) and several area of wide mesh fibrin network (asterisks). Graphene Oxide acts disrupting the fibrin network and disaggregating bacteria; in (**C**), *S. aureus* PECHA 10 cells in the GO treated specimen with damaged outer membranes (arrows) and equatorial rings (asterisks), are shown. Graphene Oxide hampers the microbial aggregation. Coccoid bacteria, characterized by plasmolysis spaces are detected.

Figure 5 displays SEM images of the GO effect on mature LCWB. Graphene Oxide demonstrates to act by disrupting the fibrin network and disaggregating bacterial cells. When compared with the untreated sample (Fig. 5A), the sample treated with GO (Fig. 5B) displays thinner and less interconnected fibrils of fibrin (asterisks). Shown in Fig. 5C is how GO also interferes on microbial clustering by producing cell dispersion. Coccoid separated bacteria, characterized by plasmolysis spaces on the outer membranes are reported and evidenced by arrows in Fig. 5C.

## Discussion

This study is focused on the search of new strategies to tackle the critical topic of antibiotic resistance in chronic wound infections. *Staphylococcus aureus* and *P. aeruginosa* are microbial species characterized by a significant multidrug-resistance and innovative plans for treatments are required^20,21^. These microorganisms represent the most frequent combined species isolated in polymicrobial wound infections and are capable to express synergism through an increased antibiotic tolerance level compared with the single species culture^5^. Evidence suggests that biofilm, together with the drug-resistant strains, plays a significant role in the inability of chronic wounds to heal. The LCWB model provides a functional system for testing antimicrobial and antibiofilm treatments, mimicking the in vivo conditions. In fact, this model is composed by blood plasma, red blood cells and *S. aureus* and *P. aeruginosa* that form a coagulated system composed by a dual species biofilm in a fibrin network, typical of the in vivo wound environment. This wound biofilm model, as reported by Brackman and Coenye^22^, is one of the most suitable and used system since it represents a realistic in vitro model, easy to handle and characterized by a rapid maturation of the multispecies biofilm.

On this 3D organized structure, the GO effect inhibiting the *S. aureus* and *P. aeruginosa* in forming and mature biofilm was evaluated.

In a previous study, we demonstrated the antimicrobial and antibiofilm effect of GO against *S. aureus* PECHA 10, *P. aeruginosa* PECHA 4 and *Candida albicans* X3 single clinical isolates with a major effect against Staphylococci^12^. This major antimicrobial action against the Gram positive bacterium was demonstrated by AFM analysis that evidenced *S. aureus* cells wrapped by GO^12,23^. In addition, as reported by Song et al*.*, GO interferes with the bacterial membrane and produces the ROS-independent oxidative stress^17^.

In the present study, in dual species LCWB model, GO, used at 50 mg/l, concentration that is demonstrated to be non-toxic for eukaryotic cells^13,24^, expresses a good microbial inhibition, confirming the major effect against *S. aureus* PECHA 10. Scanning Electron Microscopy analysis of GO treated samples demonstrates the presence of coccoid cells with depressed areas, resembling plasmolysis spaces generally observed in presence of bioactive extracts in *S. aureus* strains^25^. Deeper studies must be performed in order to evaluate the precise GO mechanism on *S. aureus* cells in LCWB model.

Interestingly, the GO antibiofilm action is meaningful also against *P. aeruginosa* in dual species biofilm when compared to the controls both in forming and mature biofilm. The GO antibiofilm effect against both in forming and mature biofilm is of interest also when compared to the AMK action that displays a marked killing action against the dual species used for the Lubbock model. On the other hand, the clinical guides on the selecting of optimal treatment plans, for chronic wound management, are focusing on novel challenges including new biocides also considering the antibiotic resistance/tolerance phenomenon^26^.

Therefore, GO can be considered a valid non-antibiotic compound useful against resistant microorganisms isolated from chronic wound.

In LCWB model, GO is capable to interfere on the *S. aureus* and *P. aeruginosa* clustering in the dual species biofilm formation and it is also capable to disaggregate and disperse microbial sessile cells in the mature biofilm. Therefore, in the first phase of microbial colonization, GO can act delaying the infection process whereas, in the mature biofilm, GO can favour the disruption of the clustered bacteria embedded in the extracellular polymeric substances promoting the release of planktonic cells for which has been demonstrated the GO effect in terms of CFU reduction^12^.

From our results, GO appears to be able to interfere with fibrin polymerization by reducing the capability of *S. aureus* to properly coagulate the system by creating an efficient and intricate fibrin network. Fibrin is a complex matrix in wound healing processes because it serves as a substrate for cell attachment, proliferation and extracellular material formation. In presence of GO, the fibrin polymerization appears full of void and loose. It is important to highlight at this stage that Ca^2+^-binding to the γ chain of fibrinogen is fundamental for modulating fibrin polymerization. Indeed, Ca^2+^-binding favours the later aggregation of proto-fibrils of fibrin to form thicker fibres. Weisel and Litvinov observed that the effect of GO is probably due to its well demonstrated ability to chelate Ca^2+^ ions thus reducing its availability in the medium and, therefore, the pathogens growth^27–30^. In addition, the interactions between the amino groups of fibrin and the carboxyl groups on edges of GO sheets, through electrostatic interactions^27^, may interfere with the fibrin polymerization. As demonstrated in the present work, by the qualitative assessments of SEM images, GO induces detachment of bacteria by degrading the fibrin scaffold holding mature biofilms together.

In recent years, graphene derivatives have attracted great interest as antibiofilm and wound healing materials^14^.

Following our data, it is possible to hypothesize that GO displays a twofold role in this scenario: (1) it reduces the *S. aureus* and *P. aeruginosa* dual species biofilm in chronic wound LCWB model; (2) it inhibits a proper polymerization of fibrin during biofilm matrix formation. The latter effect could be effectively investigated thanks to the exploited 3D LCWB.

The hypothesized effect of GO on fibrin polymerization explains also the higher effect of GO on *S. aureus* rather than *P. aeruginosa*.

In this study we demonstrate how GO affects chronic wound biofilm model such as LCWB, a model that mimics the real polymicrobial colonization of the wound in vivo. It can be concluded that GO displays an inhibitory effect on the main isolated pathogens in chronic wounds infections.

The GO double effect on microbial disaggregation and on decrease of fibrin network strongly suggest its use as antimicrobial and/or delivery system to improve the efficacy of other antimicrobial agents.

Graphene Oxide can be used to improve the wound healing, enhancing the rate of wound repair and reducing the scar formation. Future studies will be carried out to realize a wound bandages or medical devices to treat and/or prevent microbial infection.

## Materials and methods

### Bacterial cultures

Anonymized clinical strains *S. aureus* PECHA 10 and *P. aeruginosa* PECHA 4, derived from patients with chronic wounds^12^, were used in this study. These bacteria, coming from the private collection of the Bacteriological Laboratory of the Pharmacy Department, University “G. d’Annunzio” Chieti-Pescara, were cultured on Mannitol Salt Agar (MSA, Oxoid, Milan, Italy) and Cetrimide Agar (CET, Oxoid, Milan, Italy), respectively.

For the experiments, bacteria were cultured in Trypticase Soy Broth (TSB, Oxoid, Milan, Italy) and incubated at 37 °C overnight in aerobic condition and then refreshed for 2 h at 37 °C in an orbital shaker in aerobic condition. The cultures were standardized to on Optical Density at 600 nm (OD_600_) = 0.125 and diluted 1:10 for *S. aureus* PECHA 10 and 1:100 for *P. aeruginosa* PECHA 4, to obtain 10^6^ CFU/ml and 10^5^ CFU/ml, respectively.

### Preparation of Graphene Oxide aqueous dispersion

An aqueous solution of 4 g/l GO (Graphenea, Donostia San Sebastian, Spain) was added to PBS in order to reach the desired concentration, bath ultrasonicated for 10 min (37 kHz, 180 W; Elmasonic P60H; Elma), and sterilized for 2 h under a UV lamp (6 W, 50 Hz, 0.17 A; Spectroline EF 160/C FE; Spectronics). The concentration of GO was checked spectrophotometrically at λmax 230 nm. Graphene Oxide flakes dimensions were checked by using dynamic laser light scattering (DLS) (90Plus/BI-MAS ZetaPlus multi angle particle size analyzer; Brookhaven Instruments Corp.)^12^. For all of the experiments, GO was used at a concentration of 50 mg/l, concentration that has been recognized as non-toxic for eukaryotic cells and, very recently, absolutely not toxic for cutaneous administration^31–36^.

### Amikacin susceptibility assay

Amikacin was used in the experiments as positive control. The AMK MIC was performed against *S. aureus* PECHA 10 and *P. aeruginosa* PECHA 4 by microdilution method according to CLSI guidelines^37^. Amikacin was purchased from Sigma-Aldrich (Milan, Italy) and stock solution was stored in sterile water at − 20 °C. Twofold dilutions of AMK stock solution ranging from 250 to 4 mg/l were performed in Mueller Hinton Broth II cation adjusted (Oxoid, Milan, Italy). For MIC, 100 µl of AMK and 100 µl of each broth culture (10^5^ CFU/ml) were dispensed in each well of 96-wells microtiter plate and incubated in aerobic condition at 37 °C. MICs were measured by determining the lowest concentration of AMK able to inhibit the visible growth of the microorganisms.

### Chronic wound biofilm model

In this study, we used the LCWB model reported by Sun et al.^18^, with some modifications. Figure 1 displays a flow chart with a step by step clarification of the proposed experimental plan, for in forming and mature LCWBs, and GO treatment.

Briefly, 5 ml of medium containing Brucella Broth (BB, Oxoid, Milan, Italy) with 0.1% agar bacteriological, 50% porcine plasma (Sigma Aldrich, Milan, Italy), 5% horse erythrocytes (BBL, Microbiology System, Milan, Italy) and 2% Foetal Calf serum (Biolife Italiana, Milan, Italy) were distributed into glass sterile tubes. For the LCWB preparation, 10 µl of each diluted broth culture (as described above) were inoculated into glass tubes with a sterile pipette tips (Fig. 1, top). This mixture was used for the evaluation of the GO effect in forming LCWB (Fig. 1, center left). The mature modified LCWB was prepared following the Kucera et al. methodology^19^, also according to our preliminary time quantification. To define the best time to obtain mature biofilm, the biofilm biomass (volume) and the cell viability (Live/Dead staining) of the LCWB were evaluated after 24, 48 and 72 h of incubation. Forty-eight hour of incubation was the best time to obtain the most viable quantified LCWB volume that, consequently, was considered as mature biofilm. In fact, after 24 h of incubation, the biofilm biomass was less than 48 h with more planktonic microorganisms whereas, the biofilm biomass detection after 72 h produced similar biofilm biomass to 48 h with a marked dead cell component (data not shown). After 48 h of incubation, the mature biofilm was harvested from glass tube, the pipette tip was removed, and the biofilm biomass was washed two times with sterile PBS, the LCWB volumes were determined (V = π × r^2^ × h) and, then, placed on Petri dishes containing Bolton Broth (Oxoid, Milan, Italy) with 1.5% agar bacteriological to produce the “wound bed” for the chronic wound biofilm model (Fig. 1, bottom right).

### Graphene Oxide treatment in forming biofilm

For the GO effect, in forming LCWB, 100 µl of GO at a concentration of 2.5 mg/ml or 100 µl of phosphate-buffered saline (PBS) (for the control) or 100 µl of AMK (for positive control) at a final concentration of 8 mg/l (lower than MIC values of *S. aureus* PECHA 10 and *P. aeruginosa* PECHA 4) were added to the Lubbock medium. The test tubes were incubated for 48 h at 37 °C. The GO effect was determined in terms of percentage of reductions of *S. aureus* PECHA 10 and *P. aeruginosa* PECHA 4 CFU for mg of LCWB in respect to the control. After 48 h of incubation, the LCWB was harvested from the glass tube, the pipette tip was removed and the biofilm was washed two times with sterile PBS, the excess medium was removed with sterile cotton and the weight was measured. To detach clustered bacteria in the LCWB, harvested and dried biofilm was vortexed for 2 min, sonicated for 3 min (with ultrasound bath) and vortexed for other 2 min. Live/Dead staining was used to confirm the effect of this procedure in terms of disaggregating action and the cell viability retaining. The CFU/mg was determined by spreading the serial dilution on MSA and on CET and incubated at 37 °C for 24–48 h.

### Graphene Oxide treatment on mature biofilm

The GO effect on mature LCWB was evaluated following the Kucera et al. methodology^19^ as described above. After placing the mature biofilm on the “wound bed”, the LCWBs were treated with an amount of GO (at final concentration of 50 mg/l), AMK (at final concentration of 64 mg/l, greater than MIC values of *S. aureus* PECHA 10 and *P. aeruginosa* PECHA 4), and PBS (for the control) depending on each LCWB volume (V = π × r^2^ × h). The amount was determined in order to avoid the spread of the substances in the Bolton medium and to allow their totally adsorption on LCWB*.* The mean values LCWBs volumes were 0.96 cm^3^ ± 0.45, 0.84 cm^3^ ± 0.23 for GO and AMK, respectively*.* As a matter of fact, depending of the volume of the obtained mature biofilms, the amount of added GO and AMK varied in order to keep their concentrations strictly constant in all the experiments. The treated LCWBs were incubated for 24 h at 37 °C. The biofilm was harvested from the artificial wound bed by using a sterile forceps, washed twice with sterile PBS, the excess medium was removed with sterile cotton and the weight was measured. Subsequently, the biofilm was vortexed for 2 min, sonicated for 3 min (with ultrasound bath), vortexed for other 2 min and diluted in PBS for the microbial enumeration. Live/Dead staining was used to confirm the effect of this procedure in terms of disaggregating action and the cell viability retaining. The CFU/ml was determined by spreading on MSA for *S. aureus* PECHA 10 and on CET for *P. aeruginosa* PECHA 4 and the plates were incubated at 37 °C for 24–48 h. Data were expressed as CFU/mg of LCWB sample.

### Cell viability analysis

The *S. aureus* PECHA 10 and *P. aeruginosa* PECHA 4 viability in forming and mature LCWBs in presence of GO and AMK was also evaluated by fluorescence microscopy. After treatment with GO and AMK, the biofilms, as described above, were centrifuged at 10.000 rpm for 5 min and the pellet was resuspended with 10 µl of Live/Dead staining (Molecular Probes Inc., Invitrogen, San Giuliano Milanese, Italy) and visualized under a fluorescence Leica 4000 DM microscopy^38^.

Ten fields of view, randomly chosen, for each slide were examined. The determination of the bacterial viability percentage was performed independently by three microbiologists by using image analysis software (LEICA QWin, Milan, Italy)^39^. The percentage of viable bacteria was calculated as area occupied by green bacteria by using of the Image Analysis Software using the following formula for each observed field: area filled by green viable bacteria = area filled by all bacteria (both green viable bacteria and red dead bacteria) – area filled by red dead bacteria. The % of green viable bacteria was calculated assuming as 100% the total area filled by all bacteria (green viable bacteria plus red dead bacteria). Microscopic observations were repeated for three independent experiments.

### Scanning electron microscopy (SEM) observation

To evaluate the spatial microbial distribution and the Lubbock biofilm structure, the mature biofilms were fixed in a 4% solution of glutaraldehyde buffered with 0.5 M PBS to pH 7.4 overnight at 4 °C. After thorough washing with PBS, samples were dehydrated in a series of ethanol solutions at progressively higher concentration starting from 30 to 100%. Two changes (2 × 15 min) for each step were made. The samples were treated for critical point drying in Emitech K 850 (Emitech Ltd., Ashford, Kent, UK) and later were frozen in liquid nitrogen and fractured with a frozen blade to expose internal surfaces. The obtained fragments were mounted onto aluminium stubs, sputter gold coated in Emitech K 550 (Emitech Ltd. Ashford, Kent, UK) and were observed under a SEM with LaB6 electron gun (Zeiss EVO 50 XVP; Carl Zeiss SMY Ltd, Cambridge, UK) equipped with an Everhart–Thornley tetra solid-state detector (4Q-BSD). SEM operating conditions included 7 kV accelerating voltage, 8 mm working distance, and a 7pA probe current for high vacuum observations The images were captured with a line average technique using 20 scans.

### Statistical analysis

The statistical significance of differences between controls and experimental groups was evaluated using ANOVA. Probability levels of 0.05 were considered statistically significant. All data were obtained from eight independent experiments performed at least in duplicate.

## Supplementary information

Supplementary Information
