# Supplementary material for: Graphene Oxide affects Staphylococcus aureus and Pseudomonas aeruginosa dual species biofilm in Lubbock Chronic Wound Biofilm model
Source: Sci Rep. 2020 Oct 28;10:18525. doi: 10.1038/s41598-020-75086-6 (PMC7595099; doi:10.1038/s41598-020-75086-6)
Supplement: Supplementary file 1 — Supplementary Information [file 41598_2020_75086_MOESM1_ESM.docx]

**Supplementary Information**

**Graphene Oxide affects *Staphylococcus aureus* and *Pseudomonas aeruginosa* dual species biofilm in Lubbock Chronic Wound Biofilm model**

**Mara Di Giulio^1#^, Silvia Di Lodovico^1#^, Antonella Fontana^1^, Tonino Traini^2^, Emanuela Di Campli^1^, Serena Pilato^1^, Simonetta D’Ercole^2^ & Luigina Cellini^1*^**

^1^Department of Pharmacy, University “G. d'Annunzio” Chieti-Pescara, Via dei Vestini, 31, 66100 Chieti, (CH) Italy.

^2^Department of Medical Oral and Biotechnological Sciences, University "G. d'Annunzio" Chieti-Pescara, Via dei Vestini, 31, 66100 Chieti (CH), Italy.

*Correspondence and requests for materials should be addressed to L.C. (email: l.cellini@unich.it)

Mara Di Giulio^#^ and Silvia Di Lodovico^#^ contributed equally to this work

**Table S1** Dimensions of the investigated GO.

| **Sample** | **T (°C)** | **Dimensions (nm)** | **Polidispersity** |
| --- | --- | --- | --- |
| GO 50 mg/l | 25 | 598.3 ± 10.3 | 0.261 ± 0.019 |
| GO 50 mg/l | 37 | 668.1 ± 33.7 | 0.254 ± 0.013 |

**Table S2** LCWB weight (mg) ± SD and bacterial load (CFUs/LCWB mg) in untreated and GO/AMK treated samples

|  | | **LCWB weight (mg)**  **Bacterial load (CFUs/LCWB mg)** | |
| --- | --- | --- | --- |
| **Sample** | | **In forming** | **Mature** |
| *Untreated* | LCWB weight  *S. aureus*  PECHA 10  *P. aeruginosa*  PECHA 4 | 1379.35±7.42  7.83x10^3^±5.85x10^3^  3.35x10^5^±9.80x10^3^ | 595.42±49.02  3.47x10^5^±1.53x10^5^  2.52x10^5^±7.94x10^4^ |
| *GO* | LCWB weight  *S. aureus*  PECHA 10  *P. aeruginosa*  PECHA 4 | 929.30±18.24  3.52x10^3^±2.83x10^3^  1.87x10^5^±2.54x10^4^ | 451.23±34.39  1.03x10^5^±1.58x10^4^  9.14x10^4^±6.76x10^4^ |
| *AMK* | LCWB weight  *S. aureus*  PECHA 10  *P. aeruginosa*  PECHA 4 | 702.40±24.52  8.55x10^2^±3.64x10^2^  5.31x10^4^±1.29x10^4^ | 470.67±41.02  2.22x10^4^±1.56x10^4^  1.58x10^4^±1.23x10^3^ |
